# Supplementary material for: The effect of PEGylation on surface tethering of liposomes via DNA nanotechnology
Source: J Lipid Res. 2025 Nov 11;67(1):100940. doi: 10.1016/j.jlr.2025.100940 (PMC12800489; doi:10.1016/j.jlr.2025.100940)
Supplement: Supplementary Data 2 [file mmc2.pdf]

Supplementary information

**DNA strand name**	**DNA strand sequence**	**Potential DNA strand modifications**
**(1)** Toehold strand	CCTACTGACTTTATCCACCGATTCTAGGGTTAAAAGGGGACG	3’ Biotin-TEG
**(2)** Connector strand	CGTCCCCTTTTAACCCTAGAAGGGATAAGTTGATTGCAGAGC	None
**(3)** Alexa647 fluorophore modifiable strand	TCTCGACACAAATCTTCCTGCGCTCTGCAATCAACTTATCCC	3’ Alexa Fluor 647 (NHS Ester)
**(4)** Chol-DNA handle strand	GCAGGAAGATTTGTGTCGAGA	3’ Cholesterol-TEG
**(5)** Scrambled chol-DNA handle strand	CTGGTGAGACGCTAGAC	3’ Cholesterol-TEG

Supplementary Table 1. DNA strand names, sequences, and modifications. DNA strand names, sequences, and potential strand modifications that can be added to that ssDNA component to add functionality are listed.

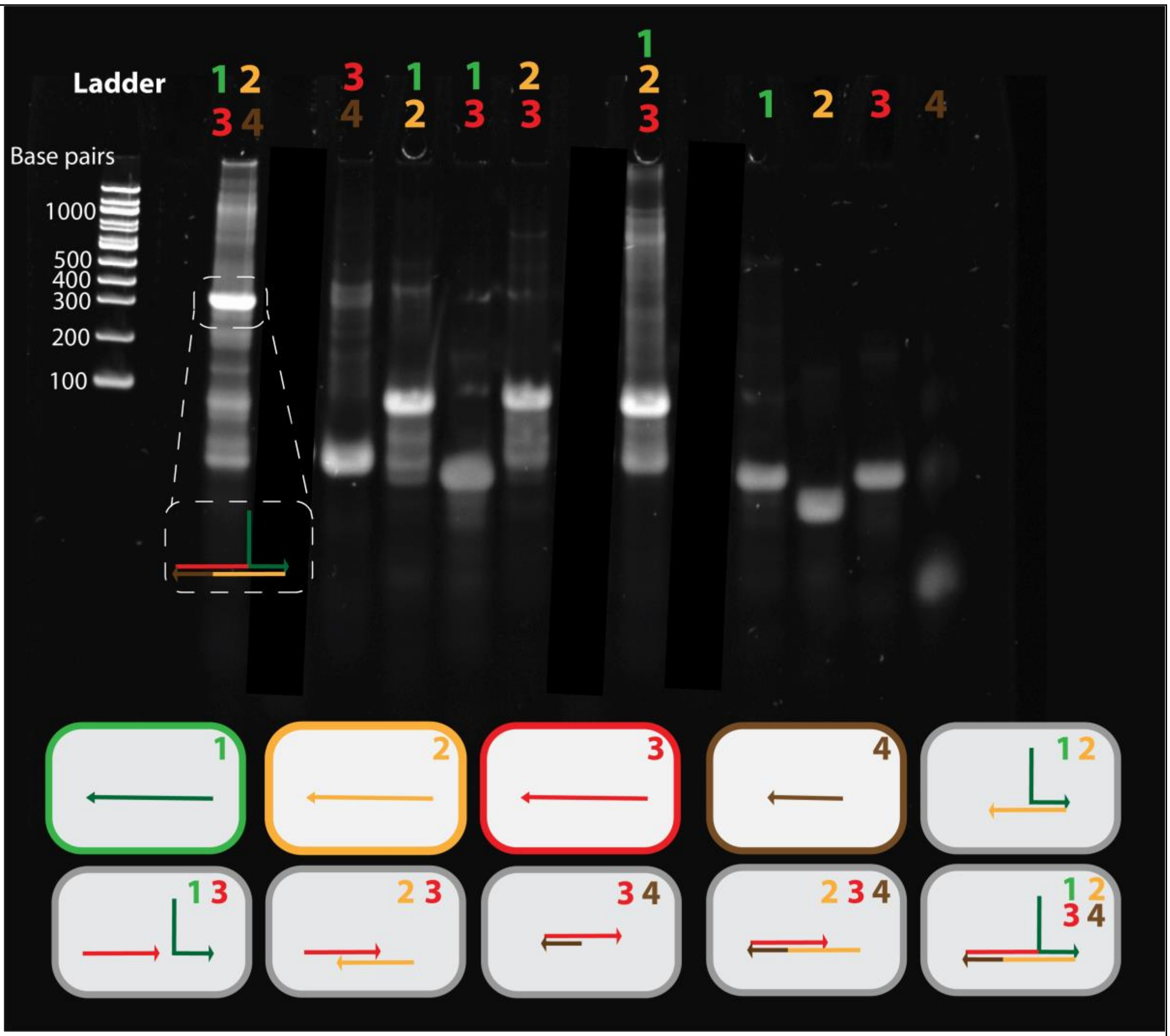

Supplementary Figure 1. 16% PAGE confirming complex formation. Individual strands and combinations present within each lane are indicated by numbers (top) corresponding to the legend (bottom). Gel visualisation of the different combinations of ssDNA leading up to the hybridisation of the full complex shows size increases that correspond to larger DNA constructs..

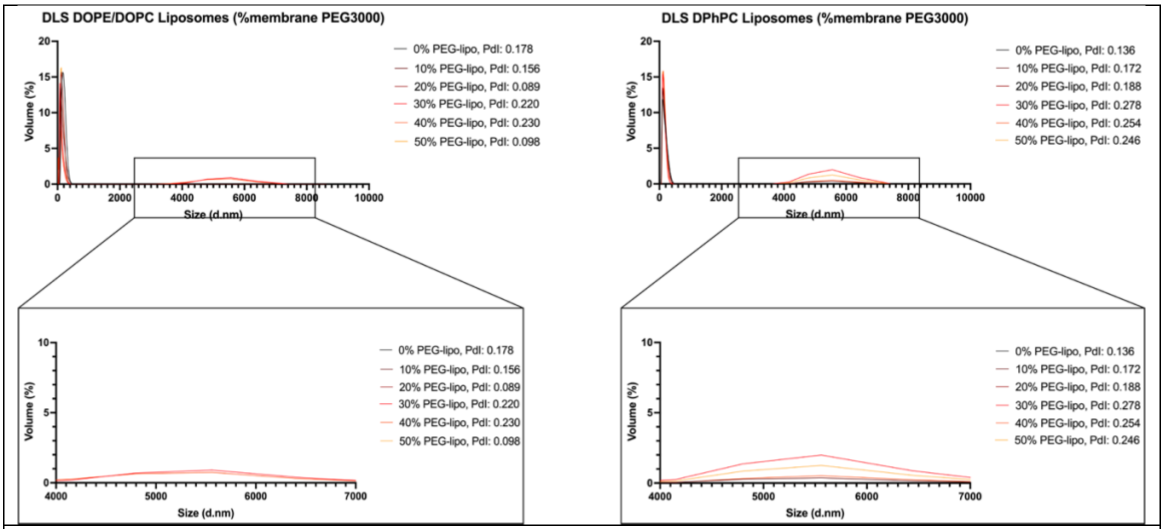

Supplementary Figure 2. DLS measurements of liposome sizes with varying amounts of PEG present in initial lipid formulations. Full DLS line plots showing size distributions for both DPhPC (right) and DOPE/DOPC (left) liposomes with 0-50% PEG3000 added to the initial lipid mix formulation. Inset graphs were displayed with different x-axes to show the secondary artefact peaks for each lipid composition (bottom). Polydispersity Index (PdI) values are also included for each condition in the figure legends.

Code available here

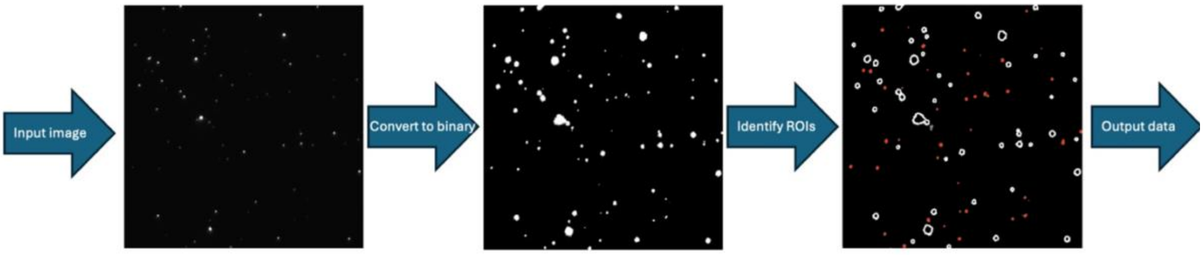

Supplementary Figure 3. Python code and graphical representation of spot detection analysis method for microscopy images. Analysis code loop was written in Python and run on Python version 3.8. Graphical representation of the image analysis pipeline (bottom) shows how images are processed from raw inputs (left) into binary images based on a minimum intensity threshold that identifies pixels associated with fluorescence ROIs to minimise background interference (middle). This is followed by ROIs being segregated into liposome spots below 50 pixels (right- red outlines) and aggregates above 50 pixels (right- white outlines) based on a chosen area threshold in order to only quantify blocking of individual liposomes as opposed to large aggregates. This fluorescence ROI data is then outputted into an excel file.

DiD labelled liposomes				
**Lipid type**	**PEG3000-PE (%)**	**Image no.**	N_{spots}	N_{agg}
**DPhPC**	**0**	#1	43	16
		#2	60	21
		#3	60	25
		#4	62	27
		#5	56	28
		**Total**		
		**5**	**281**	**117**
	**10**	#1	46	25
		#2	31	35
		#3	20	24
		#4	23	29
		#5	22	53
		**Total**		
		**5**	**142**	**166**
	**20**	#1	3	1
		#2	8	0
		#3	4	1
		#4	4	0
		**Total**		
		**4**	**19**	**2**
**DOPE/DOPC**	**0**	#1	69	8
		#2	62	8
		#3	20	6
		#4	16	1
		#5	37	6
		#6	47	26
		#7	59	13
		#8	73	17
		#9	79	11
		#10	65	19
		**Total**		
		**10**	**527**	**115**
	**10**	#1	413	37
		#2	519	75
		#3	598	48
		#4	476	134
		#5	501	106
		#6	554	89
		#7	599	115
		#8	632	64
		#9	487	36
		#10	592	67
		**Total**		
		**10**	**5371**	**771**
	**20**	#1	205	79
		#2	162	11

		#3	163	13
		#4	149	53
		#5	306	34
		#6	204	20
		#7	68	5
		#8	85	12
		#9	254	29
		#10	102	5
			**Total**	
		**10**	**1698**	**261**

Supplementary Table 2. Number of contour ROIs identified across all DiD labelled liposome conditions. For DiD labelled liposomes the number of contours was listed for liposome spot ROIs (N_{spots}) and aggregate ROIs (N_{agg}) for each image, along with the sums of total number of images and total number of ROIs for each PEG3000 liposome formulation.

**PE-Rhodamine labelled liposomes**				
**Lipid type**	**PEG3000-PE (%)**	**Image no.**	$\boldsymbol{N}_{\mathbf{spots}}$	$\boldsymbol{N}_{\mathbf{agg}}$
**DPhPC**	**0**	#1	1392	56
		#2	1121	56
		#3	1035	30
		#4	484	25
		#5	767	16
		#6	368	73
		#7	585	89
		#8	321	62
		#9	294	44
		#10	443	82
			**Total**	
		**10**	**6810**	**533**
	**10**	#1	1248	112
		#2	567	134
		#3	567	130
		#4	1030	110
		#5	1057	118
			**Total**	
		**5**	**4469**	**604**
	**20**	#1	546	24
		#2	353	23
		#3	747	8
		#4	603	24
		#5	475	22
			**Total**	
		**5**	**2724**	**101**
	**0**	#1	133	102
		#2	994	31
		#3	134	93

DOPE/DOPC		#4	293	94
		#5	170	6
		#6	500	9
		#7	350	95
		#8	212	83
		#9	257	1
		#10	1093	48
		#11	104	0
		#12	175	1
		#13	66	0
		#14	166	0
		#15	42	0
		#16	551	80
		#17	475	84
		#18	304	85
		#19	654	87
		#20	261	97
		**Total**		
		**20**	**6934**	**996**
	**10**	#1	197	83
		#2	276	83
		#3	455	56
		#4	254	86
		#5	600	81
		#6	335	190
		#7	186	153
		#8	285	141
		#9	272	141
		#10	335	172
		**Total**		
		**10**	**3194**	**1186**
	**20**	#1	213	76
		#2	343	146
		#3	199	151
		#4	232	91
		#5	318	127
		#6	373	8
		#7	454	4
		#8	163	5
		#9	103	13
		#10	150	7
		**Total**		
		**10**	**2548**	**628**

Supplementary Table 3. Number of contour ROIs identified across all PE-Rhodamine labelled liposome conditions. For PE-Rhodamine labelled liposomes the number of contours was listed for liposome spot ROIs (N_{spots}) and aggregate ROIs (N_{agg}) for each image, along with the sums of total number of images and total number of ROIs for each PEG3000 liposome formulation.

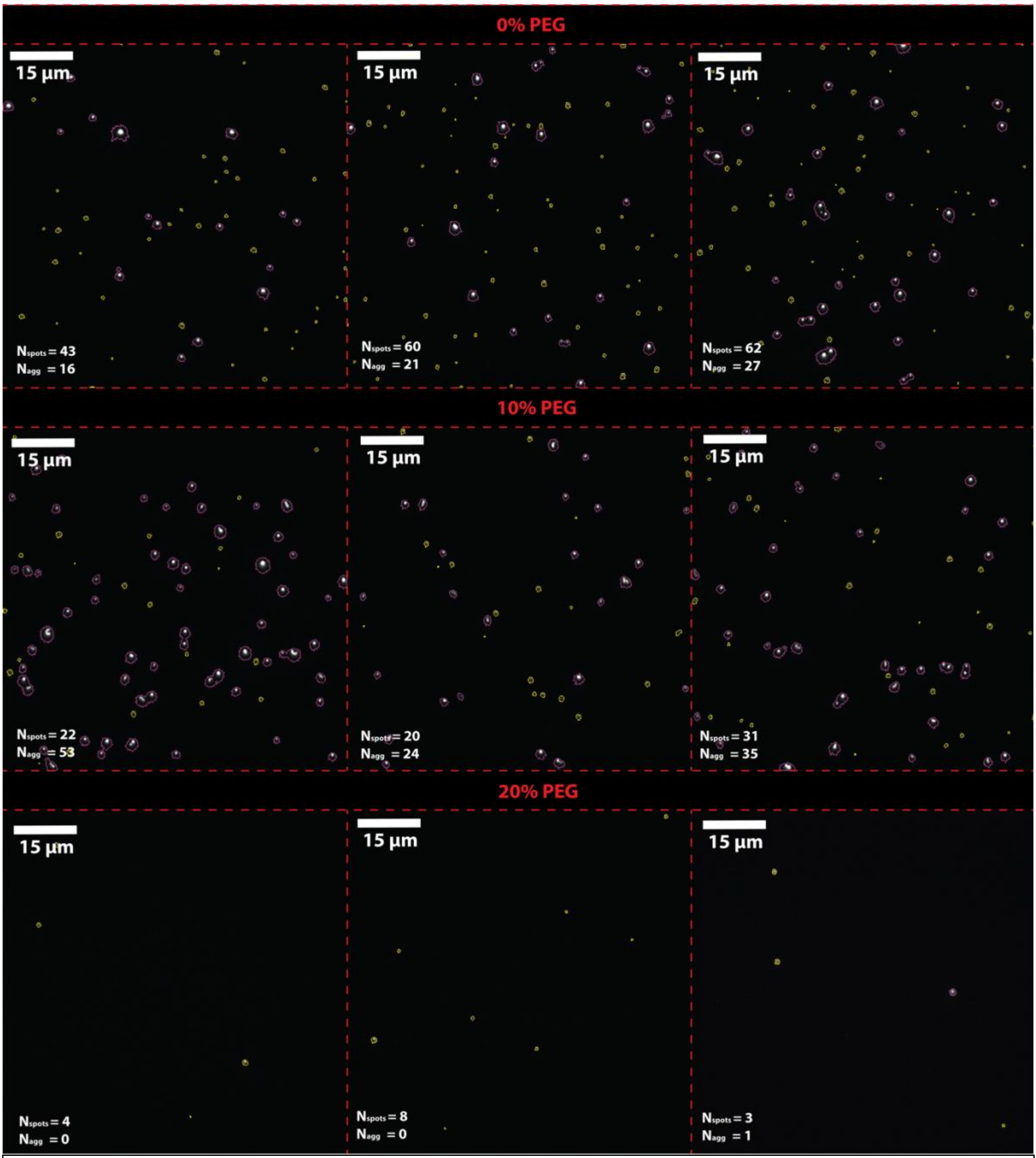

Supplementary Figure 4. Representative TIRF microscopy images of DiD labelled DPhPC liposomes. Three representative images were chosen for each PEG3000 formulation (0% top, 10% middle, 20% bottom). Liposome spot ROIs were highlighted in yellow, and aggregate ROIs were highlighted in pink. The number of liposome spot ROIs (N_{spots}) and aggregate ROIs (N_{agg}) are listed on the bottom left of each representative image.

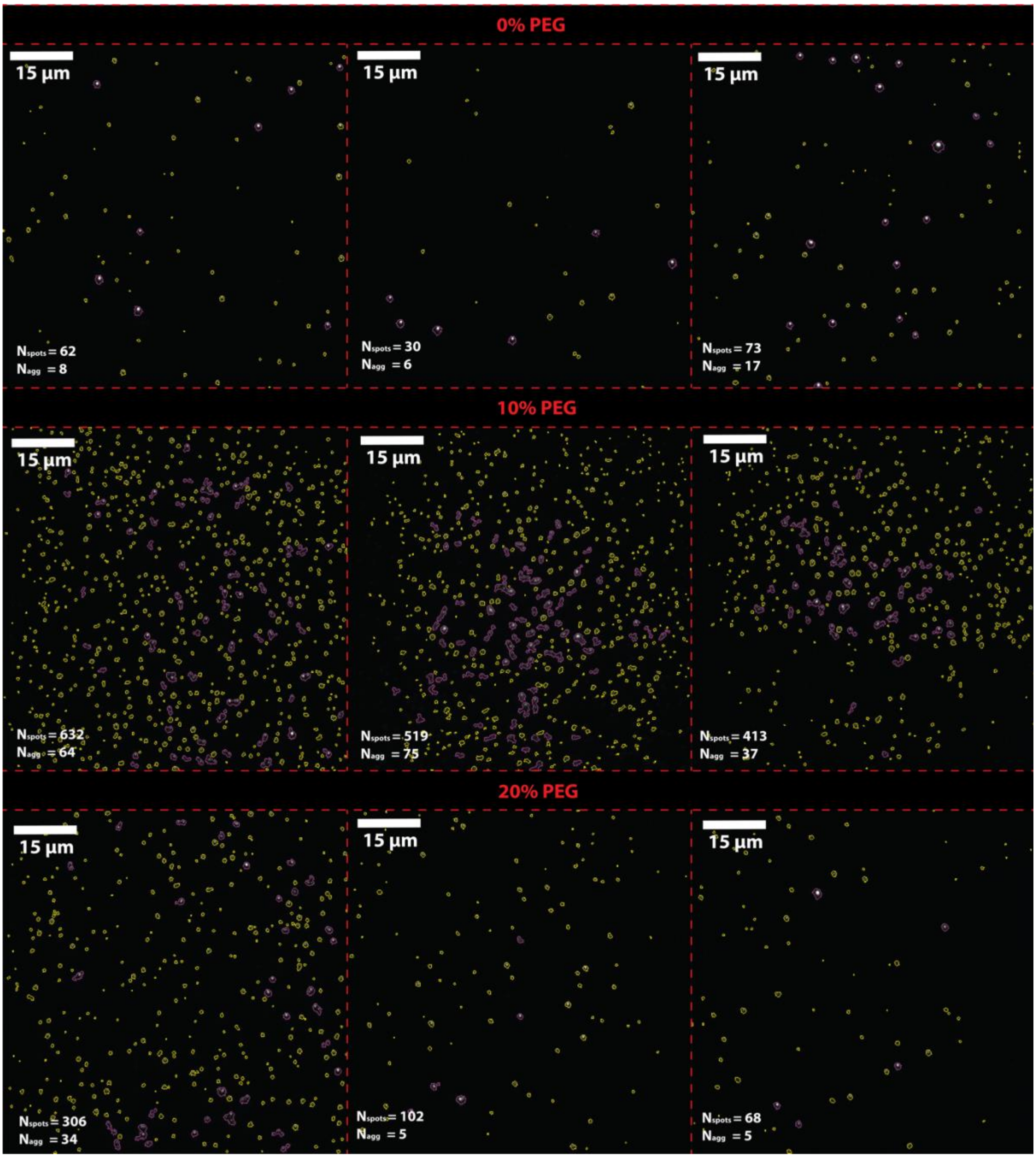

Supplementary Figure 5. Representative TIRF microscopy images of DiD labelled DOPE/DOPC liposomes. Three representative images were chosen for each PEG3000 formulation (0% top, 10% middle, 20% bottom). Liposome spot ROIs were highlighted in yellow, and aggregate ROIs were highlighted in pink. The number of liposome spot ROIs (N_{spots}) and aggregate ROIs (N_{agg}) are listed on the bottom left of each representative image.

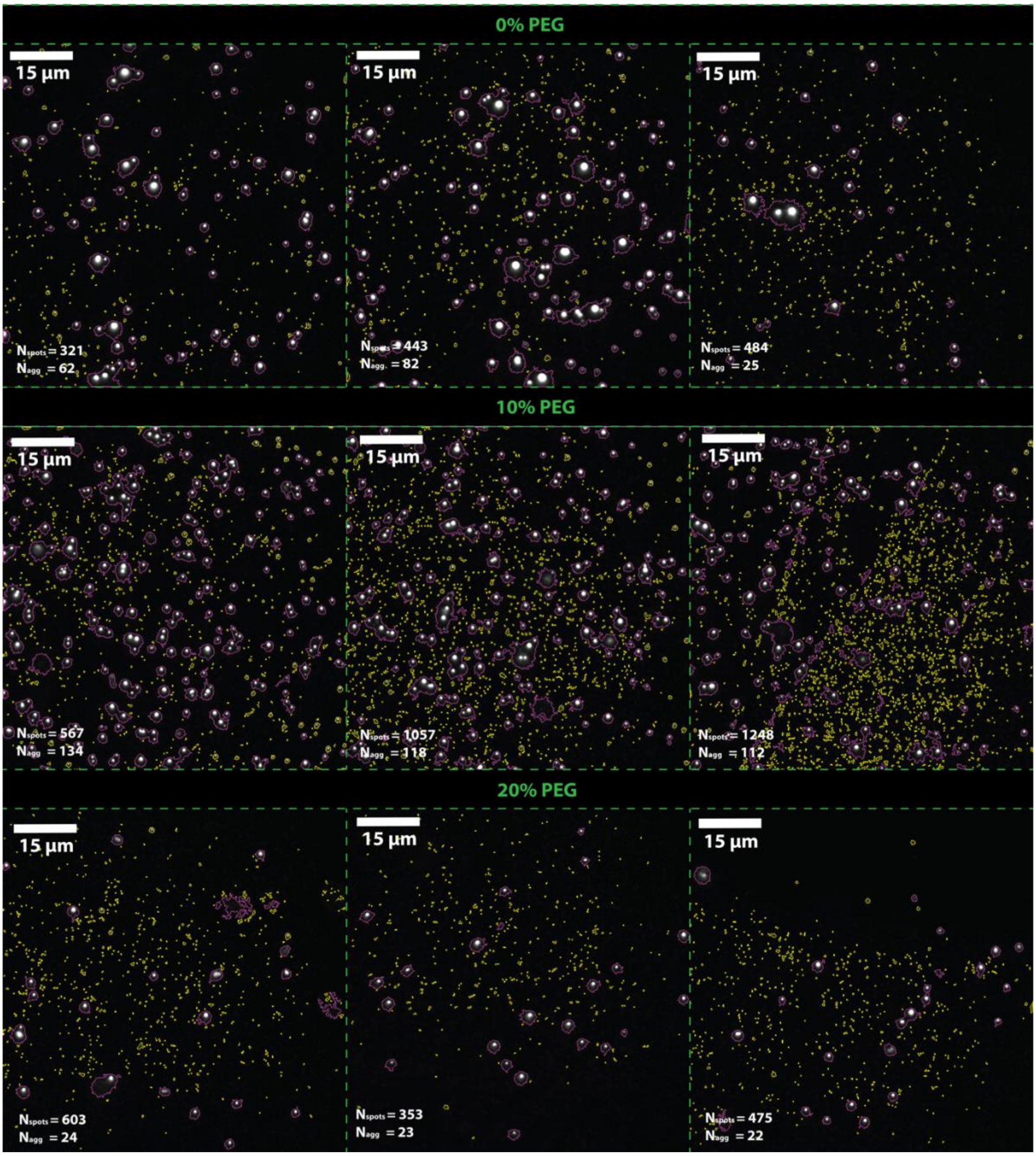

Supplementary Figure 6. Representative TIRF microscopy images of PE-Rhodamine labelled DPhPC liposomes. Three representative images were chosen for each PEG3000 formulation (0% top, 10% middle, 20% bottom). Liposome spot ROIs were highlighted in yellow, and aggregate ROIs were highlighted in pink. The number of liposome spot ROIs (N_{spots}) and aggregate ROIs (N_{agg}) are listed on the bottom left of each representative image.

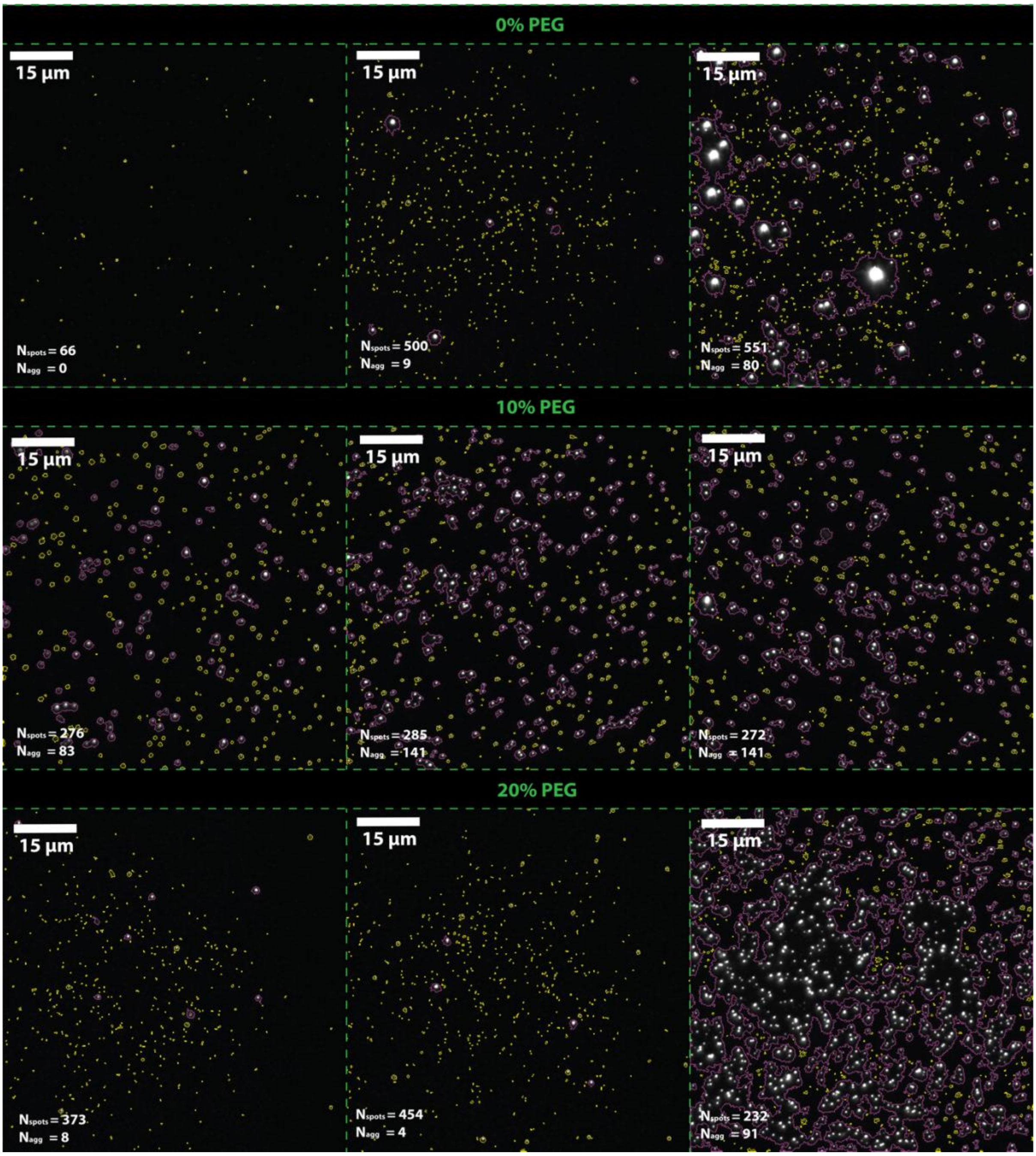

Supplementary Figure 7. Representative TIRF microscopy images of PE-Rhodamine labelled DOPE/DOPC liposomes. Three representative images were chosen for each PEG3000 formulation (0% top, 10% middle, 20% bottom). Liposome spot ROIs were highlighted in yellow, and aggregate ROIs were highlighted in pink. The number of liposome spot ROIs (N_{spots}) and aggregate ROIs (N_{agg}) are listed on the bottom left of each representative image.

DiD labelled liposomes			
**Lipid type**	**PEG3000-PE (%)**	N_{spots}	N_{agg}
DPhPC	0	281	117
	10	142	166
	20	19	2
DOPE/DOPC	0	527	115
	10	5371	771
	20	1698	261

PE-Rhodamine labelled liposomes			
**Lipid type**	**PEG3000-PE (%)**	N_{spots}	N_{agg}
DPhPC	0	6810	533
	10	4469	604
	20	2724	101
DOPE/DOPC	0	6934	996
	10	3194	1186
	20	2548	628

Supplementary Table 4. Summary of total number of contour ROIs identified across all conditions. For DiD labelled liposomes (top) and PE-Rhodamine labelled liposomes (bottom) the total number of contours was listed for liposome spot ROIs (N_{spots}) and aggregate ROIs (N_{agg}).
